# Detecting Portal Venous Blood Flow in Metabolic Dysfunction-Associated Fatty Liver Disease Using Non-Invasive Doppler Ultrasound

**DOI:** 10.7759/cureus.88446

**Published:** 2025-07-21

**Authors:** Malik Alqawasmi, Henry Lin, Nancy Kanagy, Aleksandr Birg

**Affiliations:** 1 Department of Internal Medicine, University of New Mexico, Albuquerque, USA; 2 Division of Gastroenterology and Hepatology, Department of Internal Medicine, University of New Mexico, Albuquerque, USA; 3 Department of Cell Biology and Physiology, University of New Mexico, Albuquerque, USA

**Keywords:** doppler ultrasound, hepatic circulation, metabolic-dysfunction associated fatty liver disease, metabolic dysfunction-associated fatty liver disease (mafld), portal hypertension, portal venous blood flow, steatosis

## Abstract

Introduction

Metabolic dysfunction-associated fatty liver disease (MAFLD) is the most common liver disease and is associated with metabolic syndrome, obesity, and insulin resistance. MAFLD has been shown to produce changes in portal venous blood velocity and portal pressure before the development of cirrhosis. Accurate measurement of portal venous blood flow is essential for early detection of portal hypertension and assessing fibrosis risk. In this study, we aim to evaluate non-invasive transcutaneous Doppler ultrasound versus invasive direct techniques for detecting portal venous blood flow changes in a rat model of MAFLD.

Methods

We induced MAFLD in rats by a high-fat diet (HFD). Male Sprague Dawley rats (n=16) were housed in pairs under standard conditions (12-hour light/dark cycle, temperature-controlled environment at 20-23°C) with ad libitum access to water. After a two-day acclimation period, rats were divided into standard chow diet (STD) and HFD groups (n=8 each) for 10 weeks. Portal venous blood flow was first measured non-invasively via transcutaneous Doppler ultrasound and then directly using a flow probe during terminal surgery. Additionally, peak portal venous blood flow was compared between fed and fasted states (n=3 each) using non-invasive ultrasound. Animals were randomized to the order of testing and were anesthetized prior to ultrasound using isoflurane. Each measurement was collected in triplicate by same operator and averaged.

Results

Direct measurements indicated significantly higher portal venous blood flow in HFD rats (23.4 ± 2.8 mL/min) compared to STD rats (18.0 ± 2.4 mL/min, p<0.05). However, non-invasive Doppler ultrasound did not show significant differences between HFD (13.2 ± 1.0 mL/min) and STD (12.5 ± 1.8 mL/min, p=0.74). Importantly, transcutaneous Doppler ultrasound detected a significant increase in peak portal venous blood flow in the fed state (213.9 ± 24.9 mL/min) versus the fasted state (125.5 ± 24.1 mL/min, p<0.05).

Conclusion

Non-invasive Doppler ultrasound did not detect absolute portal flow differences between HFD and STD rats but successfully identified feeding-state changes. This suggests potential use in monitoring hemodynamic changes, though further refinement and validation in larger models are needed for clinical application. Limitations include a small sample size, particularly in the fed versus fasted analysis, and the exclusive use of male rats. The accuracy of transcutaneous Doppler ultrasound was hindered by technical challenges, including difficulty imaging small vessels, operator dependency, and assumptions about vessel geometry. Additionally, non-invasive measurements significantly underestimated actual flow compared to invasive methods. These factors limit the generalizability and clinical translation of the findings, underscoring the need for further validation in larger animal models and refinement of imaging techniques.

## Introduction

Metabolic dysfunction-associated fatty liver disease (MAFLD) is a disease characterized by the presence of macrovesicular steatosis in individuals who have at least one of the following three conditions: type 2 diabetes mellitus, obesity, and metabolic dysregulation. Metabolic dysregulation is defined by the presence of at least two metabolic risk abnormalities from the following: waist circumference ≥ 102/88 cm in Caucasian men/women or ≥ 90/80 cm in Asian men/women; blood pressure ≥ 130/85 mmHg or antihypertensive medication; plasma triglycerides ≥ 150 mg/dL or triglycerides lowering medication; plasma high-density lipoprotein cholesterol (HDL-C) < 40 mg/dL for men and < 50 mg/dl for women or lipid lowering medication; prediabetes (fasting plasma glucose levels between 100 and 125 mg/dL or 2-h post-load glucose levels between 140 and 199 mg/dL or glycosylated hemoglobin (HbA1c) between 5.7% and 6.4%; homeostasis model assessment (HOMA) with insulin resistance score ≥ 2.5; and high-sensitivity C-reactive protein levels > 2 mg/L [1].

This disease is now the most common form of liver disease and is estimated to impact one-third of the U.S. population [2] and more than a billion individuals worldwide [3]. Moreover, it has been proven that the disease has a strong association with visceral obesity, insulin resistance, and endothelial dysfunction [3]. MAFLD has also been connected to metabolic syndrome, and mechanistic connections have been proposed but not yet established [4]. MAFLD is usually silent with no or subtle clinical manifestations and is discovered on labs or radiographic imaging showing steatosis [1].

The liver receives blood supply from two major blood vessels: hepatic artery and portal vein. The portal vein supplies around three-quarters of the liver’s blood, with the last one-quarter coming from the hepatic artery proper through the celiac axis [5]. Blood flow from the portal vein exposes the liver to the numerous by-products of the intestinal microbiome due to the first-pass mechanism of the liver. Some of these byproducts are toxic, such as bacterial components that induce hepatic inflammation (e.g., toll-like receptors) [6]. One proposed mechanism is through the decreased expression of cellular tight-junctions protein gene in the intestinal mucosal cells and elevated endotoxin levels in the systemic circulation, which leads to more translocation of intestinal bacteria and bacterial products into the bloodstream, predisposing the liver to more inflammatory signals [7]. An alternative mechanism proposes that the byproducts of bacterial metabolism in the gut, especially H_2_S, lower systemic blood pressure via vasodilatory effects and increase portal vein blood flow [8].

Liver cirrhosis is well recognized to alter portal venous blood flow due to endothelial dysfunction via neovascularization, extracellular matrix deposition, and enhanced contractility [3,9]. Even more, MAFLD has also been shown to produce changes in portal venous blood velocity and portal pressure before the development of cirrhosis [10,11]. Typically, portal venous blood flow is measured using invasive methods requiring catheter insertion and measurement of wedge pressures, which remains the gold standard [3]. However, non-invasive methods have been explored as an alternative typically relying on transcutaneous Doppler ultrasonography measuring velocity of the portal vein, chosen for its reliability, safety, and availability [12]. Once the velocity (V) is determined and the cross-sectional area of the portal vein is calculated using the measured diameter (D) on US, the flow rate (F) can be calculated using the following equation: F=[D/2]^2^π × V [13]. This calculation assumes uniform velocity of blood in the blood vessel and geometrically regular blood vessel shape. Despite these presumptions, the indirect method may provide an accurate estimate without requiring invasive measures. If changes in portal venous blood flow can be detected with enough sensitivity using non-invasive methods, this can help detect early cases of MAFLD and help patients at risk of developing complications. Accurate measurement of portal venous blood flow is essential for early detection of portal hypertension and assessing fibrosis risk.

To investigate this approach, we test the hypothesis that increased portal venous blood flow can be detected by transcutaneous Doppler ultrasound in rats fed a high-fat diet (HFD) [14], which has been shown to induce MAFLD in rats [15]. We also test the hypothesis that peak portal venous blood flow is increased in the fed state compared to the fasting state [14].

A portion of this research and article was previously presented as a meeting abstract and published with The American Association for the Study of Liver Diseases (AASLD) as an abstract in May of 2021.

## Materials and methods

Animals

All procedures were reviewed and approved by the Institutional Animal Care and Use Committee (IACUC) at the University of New Mexico (IACUC protocol number 200878). All protocols conformed to the National Institutes of Health guidelines for animal use in research. Male Sprague Dawley adult rats (Harlan Laboratories, Indianapolis, IN) were housed (two animals per cage) under conditions of a 12-hour light/12-hour dark cycle with environmental temperature maintained at 20-23°C and water available ad libitum. Animals were given at least a two-day acclimation prior to the beginning of any experimentation. Weight was tracked, and blood samples were collected from animals to analyze their liver functions. All data are represented as mean ± SEM.

Non-invasive versus invasive comparison

Rats were divided into two diet groups for a total of 10 weeks: HFD (n=8) (Research Diets, Inc., New Brunswick, NJ, D12451) and standard chow diet ([STD] n=8). At the end of the feeding period, intravenous blood was collected and analyzed for aspartate aminotransferase (AST), alanine aminotransferase (ALT), total bilirubin, and plasma albumin levels. After that, measurements of portal venous blood velocity were taken first indirectly using a transcutaneous Doppler probe (Vevo 3100 Ultrasound, Fujifilm, Toronto, ON), followed by invasive direct measurements (Transonic, Ithaca, NY) during laparotomy.

Before procedures, animals were anesthetized using isoflurane and were in a fasting state (16-hour fast, water ad libitum). All measurements were taken by a single operator to reduce and eliminate variability.

Non-Invasive Measurement

Mean Doppler velocity (V, cm/min) was recorded using pulse-wave Doppler mode for 3-second segments, selected based on being consistent and representative. Ultrasound was used to record the diameter of the hepatic portal vein (D) at three different time points selected based on the dimensions of the portal vein. The flow rate in the non-invasive measurements was estimated using the measured diameter (D) and the measured mean Doppler velocity (V) (Flow rate=[D/2]^2^π × V) [13].

Invasive Measurement

Following transcutaneous ultrasound measurements, animals were anesthetized again and underwent terminal laparotomy to measure direct blood flow (measured in mL/min), instrumented through a midline incision, and with direct probe access to the portal vein.

Fasted versus fed states

Three rats (from the experiment STD group) underwent repeated ultrasound measurements in fed versus fasted state to measure portal vein diameter, portal venous blood flow peak velocity, and portal venous blood flow mean velocity. Each animal underwent two non-invasive transcutaneous ultrasound sessions, one week apart, using the VEVO LAZR-X Photoacoustic Imaging System (Fujifilm). One ultrasound session was performed in the fed state (chow ad libitum until 2 hours before ultrasound), and the second ultrasound session was performed in the fasted state (16-hour fast, water ad libitum). Animals were randomized to the order of testing and were anesthetized prior to ultrasound using isoflurane. Each measurement was collected in triplicate by the same operator and averaged.

Statistics

Between-group and within-group changes in flow were compared by repeated measures two-way analysis of variance (RM-ANOVA). GraphPad Prism 9 was used for statistical analysis and graph generation. Statistical significance was defined as p < 0.05.

## Results

Baseline weight (after the eight-week diet) in the HFD group (396.6 ± 8.8 g) was significantly greater than the STD group (368.2 ± 8.0 g, p < 0.05) (Table 1). Blood sample analysis averages of the rats at the end of the eight-week period showed that rats fed HFD had AST (211.9 ± 53.6 U/L) significantly elevated compared to those fed STD (AST of 111.3 ± 28.1 U/L, p < 0.0005). 

**Table 1:** Liver function biomarkers in STD versus HFD groups Biochemical markers of liver function were measured in mice fed either STD or HFD. Data are presented as mean ± SEM (n = 8 per group). Statistical significance was assessed using a two-tailed unpaired t-test. A p-value < 0.05 was considered statistically significant. AST, aspartate aminotransferase; ALT, alanine aminotransferase; STD, standard chow diet; HFD, high-fat diet

	STD			HFD			p-Value
	Mean	SEM	N	Mean	SEM	N	
AST (U/L)	111.3	28.1	8	211.9	53.6	8	0.0004
ALT (U/L)	54.9	5.9	8	72.9	5.2	8	0.88
Total bilirubin (mg/dL)	0.188	0.04	8	0.363	0.12	8	0.99
Albumin (g/dL)	2.38	0.14	8	2.60	0.06	8	0.99

Non-invasive versus invasive comparison

**Figure 1:**
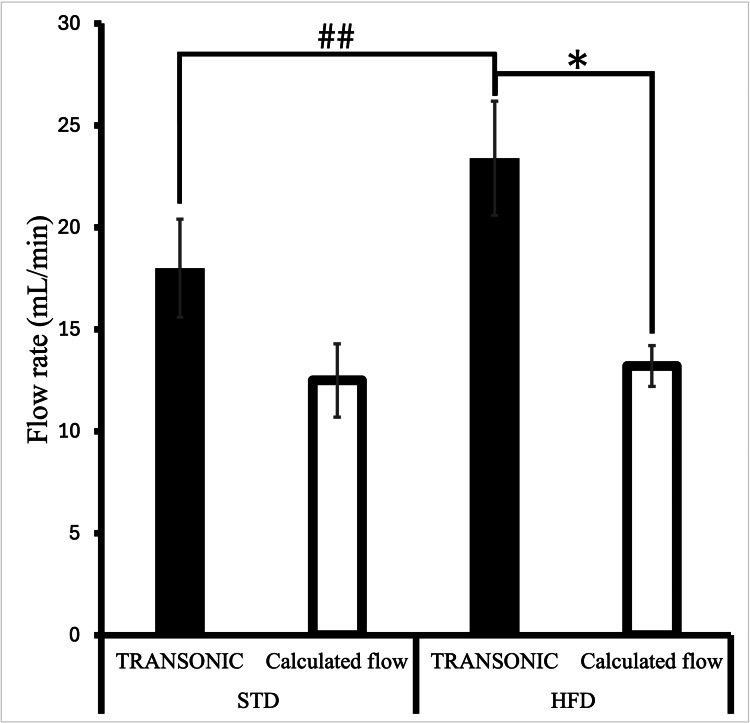
Portal venous blood flow measured in the fasted state using both methods. ##Indicates that there was a significant increase in portal venous blood flow in animals on the HFD relative to those on the STD when measured using the Transonic flow probe. *Indicates that flow estimated using the transcutaneous Doppler ultrasound was significantly lower when compared to that estimated using the Transonic flow probe. Data are presented as mean ± SEM. HFD, high-fat diet; STD, standard chow diet

When measured directly using the Transonic flow probe (invasive), there was a significant increase in portal venous blood flow in animals on HFD (23.4 ± 2.8 mL/min) relative to animals on STD (18.0 ± 2.4 mL/min) (p < 0.05) (Figure 1) [14]. Non-invasive, transcutaneous Doppler ultrasound showed no difference in flow between the HFD group (13.2 ± 1.0 mL/min) and the STD group (12.5 ± 1.8 mL/min, p = 0.74). Additionally, in HFD animals, flow estimated using the transcutaneous Doppler ultrasound (13.2 ± 1.0 mL/min) was significantly lower when compared to that measured using the Transonic flow probe (23.4 ± 2.8 mL/min) (p < 0.05) [14]. In STD animals, invasive (18.0 ± 2.4 mL/min) versus non-invasive (12.5 ± 1.8 mL/min) assessment showed no significant difference (p = 0.09).

Fasted versus fed states

**Figure 2:**
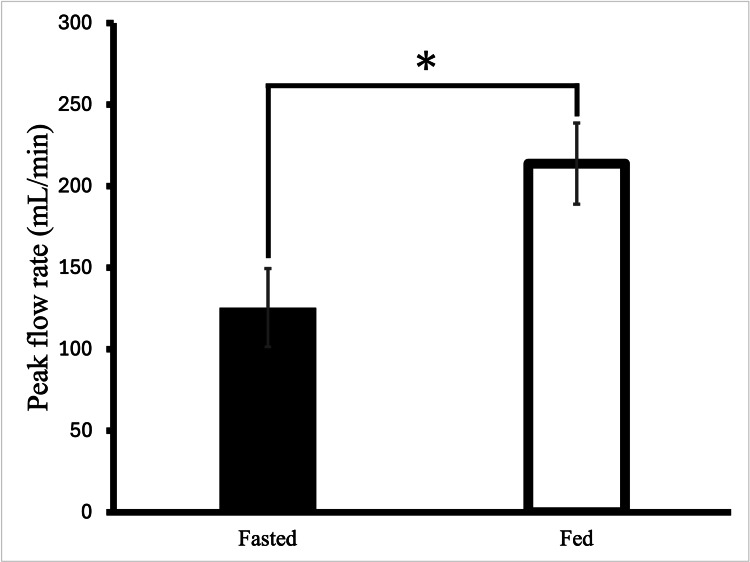
Peak portal venous blood flow in three animals in the fed and fasted states. *Shows that there is a significant increase in peak portal venous blood flow in the fed state. Data are presented as mean ± SEM.

There was a significant increase in portal venous peak blood flow in the fed state (213.9 ± 24.9 mL/min) versus the fasted state (125.5 ± 24.1 mL/min) (p < 0.05) (Figure 2) with non-invasive transcutaneous Doppler technique. However, the mean portal venous blood flow in the fed state (153.2 ± 37.7 mL/min) was not statistically different compared to fasted state (97.8 ± 10.1 mL/min, p=0.087). Additionally, there was no significant difference in the diameter of the portal vein in the fed state (2.7 ± 0.1 mm) versus fasted state (2.3 ± 0.2 mm, p=0.1).

## Discussion

In this study, we find that rats fed on a HFD had significantly elevated AST suggestive of steatosis-related changes. This is consistent with previous studies that show that HFD can induce MAFLD [5]. We also found that rats on HFD did have an elevated portal venous flow as measured directly compared to those on STD; this elevation in portal venous blood flow was not detected by the transcutaneous Doppler ultrasound. In fact, this non-invasive method underestimated portal venous blood flow compared to direct measurements. This could be explained by the reduced clarity and loss in axial resolution that occurs with ultrasound data when scanning relatively deeper objects (portal vein is deep to cutaneous tissue, subcutaneous tissue, and the liver) [16]. Another possible reason is that since the non-invasive method to measure flow is dependent on the diameter of the portal vein, scanning variabilities of the portal vein, either along different axes (not perfectly axial) or along different cross-sections, can also introduce variation. Lastly, the size of portal veins in rats is relatively small, making vein identification and proper axis orientation challenging on repeated measurements. This may not be a significant issue in clinical studies with larger measured portal veins. Size of the portal vein may also not drastically change prior to developing fibrosis [17]. Overall, this emphasizes the fact that ultrasound measurements are not an adequate non-invasive substitute for the gold standard of pressure measurements using hepatic vein catheterization in animal studies [18]. With that said, recent novel techniques have been explored to utilize endoscopic ultrasound (EUS)-guided venous pressure measurements using fine needle aspiration of the portal vein in human clinical testing [19]. Given that EUS is significantly closer in proximity to the portal vein and the invasive nature of needle-guided EUS measurements, EUS-guided non-invasive transcutaneous Doppler should be explored.

The non-invasive method was able to detect increased peak portal venous blood flow in the fed state compared to the fasted state. While this animal model evaluated non-fibrotic liver without portal hypertension, measuring relative changes in blood flow in post-prandial states in healthy livers can be a useful tool in future studies. Postprandial hyperemia has been documented in cirrhosis [20], and measurable differences in prandial states, as seen in this study, can be indicative of abnormal blood flow in fatty liver disease as an early sign of liver damage.

Additionally, our results suggest the possibility that a meal-driven surge in portal venous blood flow may exceed vascular accommodation, leading to a detectable rise in peak but not mean portal venous blood flow. While an increase in splanchnic and portal blood flow may be mitigated by a normally compliant vascular system, a meal-driven surge in portal blood flow in cirrhosis (when intrahepatic vascular resistance is increased) may lead to an even greater rise in portal pressure in the fed state. This increase in resistance and decrease in compliance is also suggested by Balasubramanian et al. and Solhjoo et al. [12,21].

In terms of limitations, this study is limited by its small sample size and the use of a single animal model, which may not fully recapitulate human MAFLD pathology, while also not having histologic or radiographic confirmation of MAFLD or hepatic steatosis. In addition, transcutaneous Doppler ultrasound assessments are inherently operator-dependent and may suffer from measurement variability, particularly in small animals. Additionally, there was no blinding, which can introduce bias. The inability to measure absolute flow accurately in MAFLD versus control groups using non-invasive methods limits direct clinical translation. Lastly, no invasive measurements were completed for fed versus fasted animals, making validation of the non-invasive measurements an important prospective goal. Future studies should evaluate refined imaging modalities or larger animal models to validate these findings.

Our findings emphasize that non-invasive transcutaneous Doppler ultrasound, while promising for detecting physiological changes such as postprandial portal flow elevations, remains limited in accuracy and clinical translatability when used in small animal models with complex pathologies such as MAFLD. Measuring and monitoring peaks in portal venous blood flow can provide an important methodology to reduce consequences of portal hypertension through monitoring dietary changes that lead to elevated peaks in portal venous blood flow [21]. The safe and non-invasive nature of this procedure makes it ideal for early detection of possible consequences of elevated portal venous blood flow. This method is also relatively low in cost and non-intrusive to patients, which can reduce foreseeable barriers [18].

It is important to mention that this elevation in peak portal venous blood flow was not detected, with statistical significance, in fasted rats on HFD compared to rats on STD. With that said, the elevation in peak portal venous blood flow in fed rats compared to fasted rats suggests plausibility to the claim that non-invasive transcutaneous Doppler ultrasound can detect elevations. Moreover, such elevations may be the underlying reason behind a portion of hepatic complications [20].

## Conclusions

In summary, our findings show that while non-invasive transcutaneous Doppler ultrasound is limited in detecting absolute portal venous flow differences in MAFLD, it can effectively capture dynamic changes in response to feeding. This suggests potential utility for monitoring physiologic states such as postprandial hyperemia. However, technical limitations, including low imaging resolution in small animals, challenges in consistent vessel alignment, and assumptions about vessel geometry, reduce its accuracy. Additional constraints of this study include the small sample size and operator dependency. Despite these limitations, further advancements in imaging resolution and standardized techniques may enhance its role in non-invasive hepatic assessment.
